# The validity and reliability study of the Turkish version of engagement in healthy ageing

**DOI:** 10.55730/1300-0144.5351

**Published:** 2022-12-04

**Authors:** Ramazan KIRAÇ, Seda UYAR, Mehmet Akif ERİŞEN, Nur Feyzal KESEN

**Affiliations:** 1 Healthcare Management, Faculty of Economics and Administrative Sciences, Kahramanmaraş Sütçü İmam University, Kahramanmaraş, Turkey; 2 Healthcare Management, Faculty of Medicine, İstanbul Medeniyet University, İstanbul, Turkey; 3 Healthcare Management, Faculty of Medicine, Selçuk University, Konya, Turkey; 4 Social Service, Faculty of Medicine, Selçuk University, Konya, Turkey

**Keywords:** Elderliness, engagement in healthy aging, validity, reliability

## Abstract

**Background/aim:**

A long life to be spent in a healthy, independent and vigorous way is one of humanity’s biggest dreams. This is the engagement of individuals in healthy ageing processes. This study was carried out to conduct the Turkish validity and reliability study of the “Engagement in Healthy Ageing Scale” developed by Menichetti, Bonanomi, and Graffigna [3] to determine the engagement of individuals in healthy ageing and their experiences with healthy ageing.

**Materials and methods:**

The quantitative research design was used in the study and descriptive findings were presented. Personal information form and the “Engagement in Healthy Ageing Scale” were used in the study’s data collection. In addition, the “Health Seeking Behaviour Scale”, and the “Self-Efficacy Scale” were used to conduct context validity. The data of the study were analysed with the help of SPSS and the LISREL package program.

**Results:**

It was determined that the goodness of fit index values of the Engagement in Healthy Ageing Scale, which was adapted into Turkish, showed good fit and acceptable fit. To test the context validity, the correlational relationship of the Engagement in Healthy Ageing Promotion Scale with the Health Seeking Behaviour and Self-Efficacy scales were examined. As a result of the correlation analysis, it was determined that there is a positive relationship between engagement in healthy ageing and health-seeking behaviour and self-efficacy.

**Conclusion:**

As a result of the research, it was determined that the Engagement in Healthy Aging Scale is a valid and reliable measurement tool in Turkish culture (Appendix).

## 1. Introduction

Ageing is a process that is impossible to be prevented, it is a process that has got chronological (according to the date of birth), biological (with anatomical and physiological changes), economical, social (the role of the elderly in life), psychological dimensions and problems that all living individuals will certainly experience [1,2]. Societies around the world are ageing and life expectancy in Europe has increased by nearly 10 years in the last 50 years, and estimates are that this increase will continue steadily and continuously over the coming decades [3]. Longer life is very valuable to people [4]. It provides individuals with an opportunity to reevaluate not only what old age might be, but how the rest of their life might develop. Moreover, as younger individuals begin to expect a longer life, they may plan their lives differently [5]. However, although the increase in longevity is pleasing, especially for elderly people who do not feel independent, active and healthy during their lifetime, this may prevent them from getting satisfaction from life [6]. Therefore, it is important that the life expectancy is increased, but what is more important here is that the increased life years are spent healthily. So much so that if people live these years in good health, their ability to do the things that matter to them will differ from that of a young person. However, if declines in physical or mental capacity predominate in this extended life span, the outcomes for the elderly and society are much more negative [5]. In this sense, the concept of healthy ageing comes to the fore.

Healthy ageing in the growing ageing population is becoming an important factor for reducing the burden of disease and disability and associated health costs [7]. Healthy ageing is a multidimensional concept and it includes not only the absence of clinical disease but also preventing physical disability and preserving cognitive, emotional and social functioning [7,8]. There are several conceptual and measurement challenges in the field of healthy ageing. One of the biggest challenges is the lack of an agreed conceptual framework [4,9]. In the study conducted by Cosco et al. [10], it was found that there were 105 definitions regarding healthy ageing. 92.4% (97) of these definitions included physiological function (e.g., physical function), 49.5% (52) included the engagement status (e.g., volunteering), 48.6 (51) included well-being status (e.g., life satisfaction), 25.7% (27) included personal resources (e.g., resilience) and 5.7% (6) included external factors (e.g., finance) [10]. According to the definition of Rowe and Kahn, “the healthy elderly” are the group with “low probability of disease and disability, high cognitive and physical functional capacity and an active relationship with life” [11]. In the broadest sense, it has been stated that healthy ageing relates to the “process of optimising physical, social and mental health opportunities so that the elderly can take an active part in society without discrimination and enjoy an independent and quality life” [12,13]. World Health Organisation defines healthy ageing as “the process of developing and maintaining the functional ability that enables well-being in advanced ages”^1^. The definitions used are generally based on two different theoretical perspectives. The first refers to the biomedical model of ageing supported by the psychological dimension and social activity along with the importance of physical health, functional and cognitive capacity [14]. The second focuses on psychosocial dimensions of healthy ageing which emphasise personal well-being and are gained through socialisation [9], such as looking for new opportunities to enjoy life at advanced ages, participation in different social environments, psychological well-being and social activities performed by the elderly, regardless of physical health [14]. All of these definitions briefly express healthy and prosperous ageing in every sense.

The concepts of health-seeking behaviour and self-efficacy are considered as concepts related to the healthy aging of individuals. Health-seeking behaviour can be evaluated from two perspectives as health care seeking behaviour and health-seeking behaviour. Health care seeking behaviour can be considered as the behaviour of individuals about where to apply in the health system, while health-seeking behaviour can be considered as the behaviour of people about what they do when they feel unwell [15]. From this point of view, the concept that is thought to be related to participation in healthy aging is the health-seeking behaviour of individuals rather than the health care seeking behaviour. On the other hand, in the study conducted by Gözüm and Aksayan [16], it was emphasized that the concept of self-efficacy, which expresses seeing self-competent, is closely related to health behaviours. Since participation in healthy aging is thought to be related to these two concepts, the relationship between health-seeking behaviour and self-efficacy and participation in healthy aging was also examined in order to test the context validity in the study.

The health outcomes of the society are shaped around the interactions between individuals and the various physical, social and political contexts (including the environment, social supports and relationships, attitudes, services, systems and policies) [17]. Therefore, the key element of the concept of healthy ageing consists of a set of health behaviours; these can be listed as modifiable behavioural factors directly related to maintaining health in older adults, such as smoking status, physical activity level, diet and alcohol use, as well as various health practices [18–20]. In a systematic review of the evidence on the behavioural determinants of healthy ageing, Peel et al. [19] confirmed that healthy ageing is associated with not smoking, being physically active, maintaining a normal weight and moderate alcohol consumption [13]. Inactivity also causes various health and functional problems in old age and has significant effects on strength, flexibility, aerobic capacity, walking capacity, balance and mental and cognitive decline [7]. Based on all these, healthy ageing is possible by early diagnosis and treatment of chronic diseases that may arise, regulating the socioeconomic conditions that may affect the health of the elderly, developing healthy behaviours among the society and the elderly, and making the necessary arrangements to create a safe and healthy environment for the elderly [21,22]. Of course, the issue that is more important than providing these opportunities is ensuring the engagement of individuals in healthy ageing processes. In a definition, healthy ageing is expressed as a lifelong process that optimizes health and opportunities to improve and maintain physical, social and mental health, independence, quality of life, and foster successful life-flow transitions. This definition describes healthy ageing as a complex process of adaptation to physical, social and psychological changes throughout life [23]. In addition, the basis of healthy ageing is the individual’s taking a role in decisions about their own life. An individual who spends healthy ageing processes efficiently minimizes the negative effects that may arise during the ageing period and minimizes the need for others until the moment of death [24]. Based on these definitions, it is possible to say that healthy ageing is not only a process that covers advanced ages but also the whole life of the individual. Therefore, engagement in healthy ageing should be provided over all age groups as a part of the whole life rather than a specific age group of the population [25].

In this context, when the national literature is examined, there is a successful aging scale, which is thought to be associated with healthy aging and was adapted into Turkish by Hazer and Özsungur [26]. However, this scale is about healthy lifestyle, adaptive coping and commitment to life rather than healthy aging. In addition, the Turkish version of the scale can be applied to individuals over the age of 60. Although the scale of attitude towards elderliness and aging, developed by Otrar [27] on individuals over the age of 18, covers not only the elders but also all adult individuals, this scale is intended to measure individuals’ attitudes towards aging rather than healthy aging. It includes dimensions such as difficulty in accepting old age, perception of social wear, difficulty in coping with life and negative image. In addition, when the literature on elderliness is examined, there are studies such as the adaptation of the geriatric depression scale [28], healthy lifestyle behaviours and related factors in the elderly [29], and a theoretical view on the relationship between active aging and lifelong learning [30]. However, there is no measurement tool that covers all adult age groups and aims to measure the engagement in healthy aging of individuals. Therefore, it is thought that the scale, which was adapted into Turkish in this study, will provide ease of application to researchers since it is a short form with eight questions, as well as make a significant contribution to the literature.

The tendency and attitude of individuals to be involved in healthy ageing processes is an important factor that can explain health-promoting behaviours [3]. The idea of healthy ageing, which also affects many economic, social and cultural factors, is a multidisciplinary situation in which the individual is at the centre. Many positive outcomes such as reducing dependency in advancing ages, active participation in the labour market and participation in society can be achieved through healthy ageing. Therefore, with a measurement tool that can determine the engagement of individuals in healthy ageing and their experiences with healthy ageing, the level of participation of the society in a healthy life has been determined, and it also functioned as a guide for the necessary regulations policies. In addition, it is thought that the inclusion of such a measurement tool will increase the awareness of both professionals and advanced adults before they enter old age, and as a result, the measures taken, the policies to be created and the services provided will contribute to the high quality of life of the elderly. In this context, it was aimed to adapt the “Engagement in Healthy Ageing Scale” developed by Menichetti et al. [3] to the Turkish culture.

## 2. Materials and methods

This study aimed to apply the Turkish validity and reliability of the “Engagement in Healthy Ageing Scale” developed by Menichetti, Bonanomi and Graffigna [3]. In the research, the quantitative research design was used, and descriptive findings were presented. In the simplest terms, quantitative research is the study that requires the collection and analysis of quantitative data.

The study was applied to individuals living in Kahramanmaraş city centre in 2020. A total of 654 thousand people live in the centre, 70% of which are adults. To determine the sample size, the table showing the acceptable minimum sample sizes for certain universes created by Coşkun, Altunışık, Bayraktaroğlu, and Yıldırım [31] was used. It was planned to include 382 people in the study sample, but 251 people were reached due to the pandemic.

Personal Information Form and “Engagement in Healthy Ageing Scale” were used in the study’s data collection. In addition, the “Health Seeking Behaviour Scale” developed by Kıraç [32], and the “Self-Efficacy” scale developed by Sherer et al. [33] and adapted into Turkish by Gözüm and Aksayan [17], were used to conduct context validity. The data of the research were analysed with the help of the SPSS and the LISREL package programs.

Following correspondence with the scale owner, the ethics committee approval was obtained from “T.C Kahramanmaraş Sütçü İmam University Rectorate Social and Human Sciences Ethics Committee” on 30.12.2020 with number E-72321963-020.

“Engagement in Healthy Ageing Scale” consists of one dimension and 8 items in total. The items of the scale were prepared with the Likert method and continued as follows, 1 “Strongly disagree”, 2 “Disagree” 3 “Undecided” 4 “Agree” 5 “ Strongly Agree”. The scale has no cut-off points. The scores obtained from the scale showed that the engagement in healthy life increased when the number got closer to 5, and the engagement in healthy life decreased when the score got closer to 1. In the validation phase of the scale, first language and content validity, then structure and context validity were made. Language and content validity is performed to determine to what extent the items of the scale represent the situation to be measured [34]. Experts in the field make judgments about the representativeness of the scale. Based on these judgments, a conclusion is reached about the validity of the scale [35].

In the first stage of the study, language and content validity were tested using the translation-back-translation method. the “Engagement in Healthy Ageing Scale” was first translated into Turkish by two English linguists. These translations were then converted into a single Turkish form most appropriately by a different person who has a good command of English and Turkish. Afterwards, this Turkish form was translated back to English by an English linguist who was not involved in the other stages of translation. After comparing the expressions in the English translation of the scale with the original English expressions, the Turkish translation was revised. As a result of this comparison, the Turkish version of the scale was found to be compatible with the original scale. Finally, the scale was presented to three field experts for content validity. While evaluating the suitability of the items, experts were asked to give each statement a score between (1) “Not accurate, should be removed“ and (4) “Completely accurate”, and the scores obtained were subjected to Kendall’s test. It was determined that there was no significant difference between the scores obtained (p > 0.005, W = 0.211, n = 7).

In the second stage of the study, a structural validity analysis was performed. Construct validity indicates the degree to which a test can accurately measure an abstract concept in the context of the behaviour to be measured [36]. The method to be used to test the structural validity of a scale is the factor analysis [37]. Factor analysis is divided into two as exploratory factor analysis (EFA) and confirmatory factor analysis (CFA) [38].

Confirmatory factor analysis was used to determine the construct validity. In addition, for context validity, correlation analysis was conducted between the “Engagement in Healthy Ageing in Scale” and “Health Seeking Behaviour Scale” and “Self-Efficacy Scale”. To determine the invariance of the study concerning time, test-retest analysis was performed one month later.

## 3. Findings

**Figure 1:**
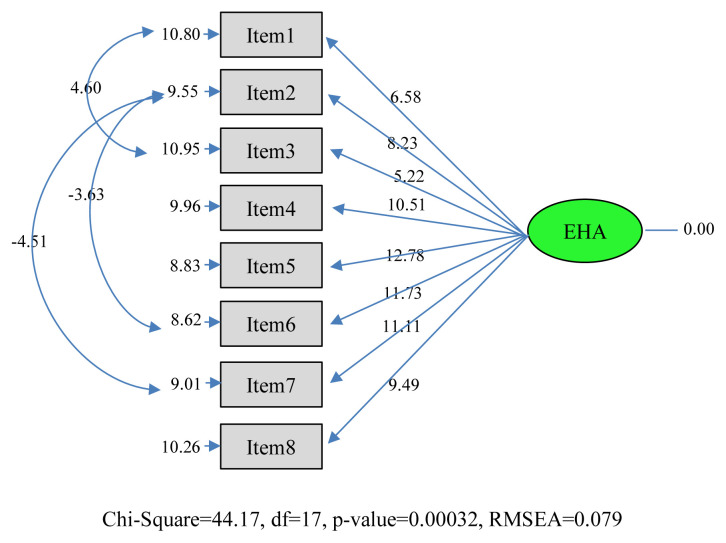
Engagement in Healthy Ageing Scale confirmatory factor analysis path diagram (t values).

**Figure 2:**
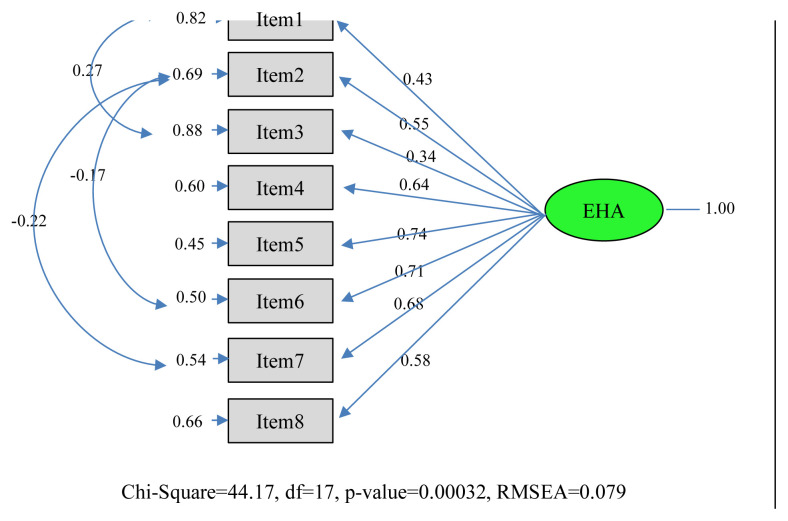
Engagement in Healthy Ageing Scale confirmatory factor analysis path diagrams (standard coefficients).

In the findings section of the study, demographic data (Table 1) and t values of the confirmatory factor analysis path diaphragm of the “Engagement in Healthy Ageing Scale” and coefficient values are given (Figures 1 and 2). Then correlation analysis findings are included for context validity. Finally, test-retest analysis is included (Tables 2 and 3).

Table 1 shows the demographic findings of the participants. Accordingly, 32.3% of the participants are 18–30 years old, 61% are women, 63.3% are married, 39.4% are university graduates, and 73.3% are middle-income. In addition, 76.9% of the participants have a nuclear family structure, 80.5% have a chronic disease, and lastly, 76.9% use a drug continuously.

The t values of the scale items are given in Figure 1. In line with the analyses made, it was seen that the level of representing its latent variable of all items in the factors (observed variable) was significant at the 0.05 level. The t values calculated for the 9 items specified are greater than 1.96, which is the critical value determined for the 0.05 significance level. In addition, the coefficient values of the scale are as in Figure 2. Accordingly, it was seen that the scale was well represented by the items.

In Table 2, the goodness of index values of the scale and normal and acceptable goodness of fit index values are given. Accordingly, it is seen that the goodness of fit values of the scale show good fit and acceptable fit. It is seen in the literature that these values are in the acceptable range [39–45].

The Cronbach alpha coefficient is used to measure the internal consistency of the scales. The Cronbach alpha coefficient indicates whether the scale items have a homogenous structure. The Cronbach alpha value used in the Likert-type scales indicates that values between 0 and 0.40 show low reliability, values between 0.60 and 0.80 show moderate reliability, values between 0.80 and 1.00 show high reliability [46]. As seen in Table 3, the item-total correlation analysis of the scale was performed. The overall reliability of the scale was found to be 0.800. This result indicates that the scale has a high level of reliability.

As seen in Table 4, a correlation analysis was performed between the Health Seeking Behaviour Scale and the Self-Efficacy Scale to carry out the context validity of the Engagement in Healthy Ageing Scale, a positive correlation was found between the scales (p < 0.001) [47]. As individuals’ participation in healthy ageing increases, health-seeking behaviour and self-efficacy also increase.

### 3.1.Test-retest analysis

In the Pearson product-moment correlation analysis, which shows the compatibility between the test-retest mean scores of the scale, a statistically significant and positive correlation was found between the two measurements (r = 0.600; p = 0.001). The difference between the scores obtained with the results of two measurements of the scale repeated with a one-month interval was examined using t-test analysis in dependent groups and it was determined that the difference between the two applications was not statistically significant (t = −0,074; p = 0.825).

## 4. Discussion

The aim of this research is to adapt the measurement tool developed in Italy in order to measure the participation of individuals in healthy aging to the Turkish language. When the literature was reviewed, it was determined that the scale had not been adapted in any country before. In addition, it was confirmed that the scale was not adapted to other languages by contacting the authors who developed the scale. The validity and reliability of both scales were examined. The scale, which was developed for the elderly in Italy, was applied to adults in Turkey. As a result of confirmatory factor analysis in both scales, it was determined that goodness of fit values showed good and acceptable fit. Although there were measurement tools such as healthy lifestyle behaviour, successful aging and active aging in Turkey, there is no healthy aging scale that includes all individuals. By adapting this measurement tool, it will be possible to measure the emotional, cognitive and behavioural tendencies of individuals to participate in healthy aging throughout their entire lives. The scale of participation in healthy aging adapted to Turkish culture is original and has the quality to contribute significantly to the literature. In addition, professionals and academics who will use this scale will be able to measure the participation of individuals in the healthy aging process and evaluate the factors affecting this process. Participation in healthy aging can be considered alone in studies to be conducted in this area. It is also recommended to examine the relationships between participation in healthy aging and issues such as health-seeking behaviour, self-efficacy, healthy living skills, self-neglect behaviour in the elderly, and attitudes towards aging. The research has some limitations. Since the sampling method used is a purposeful convenience sampling, it cannot be generalized to the Turkish population. It should also be taken into account that the data were collected during the pandemic.

## 5. Conclusion

This study aimed to test the Turkish validity and reliability of the Healthy Ageing Scale developed by Menichetti et al. [3]. In this context, the language and content validity, construct validity, and context validity of the relevant scale were tested, respectively. Finally, the relationship and differences between the two measurement averages of the scale were tested with the test-retest method. After evaluating the expert opinions the language and content validity of the scale, confirmatory factor analysis was performed to test the construct validity As a result of confirmatory factor analysis, it was determined that the goodness of fit index values of the Engagement in Healthy Ageing scale, which was adapted into Turkish, showed good fit and acceptable fit. To test the context validity, the correlational relationship of the Engagement in Healthy Ageing with the Health Seeking Behaviour and Self-Efficacy scales were examined. As a result of the correlation analysis, it was determined that there is a positive relationship between engagement in healthy ageing and health-seeking behaviour and self-efficacy. Finally, in the test-retest analysis, it was revealed that there was a significant positive correlation between the means of two measurements (r = 0.600; p = 0.001) and there was no significant difference between the means (t = −0,074; p = 0.825). As a result of the analyses made, it was determined that the Engagement in Healthy Ageing Scale developed by Menichetti et al. [3] is a valid and reliable measurement tool in Turkish culture (Appendix).

## Figures and Tables

**Table 1:** Demographic data of participants in the study.

		Number (n)	Percent (%)
**Age**	18–30	81	32.3
	31–40	60	23.9
	41–50	44	17.5
	51 years old and above	66	26.3
Sex	Male	98	39.0
	Female	153	61.0
**Marital** s**tatus**	Married	159	63.3
	Single	78	31.1
	Divorced	10	4.0
	Living separately	4	1.6
**Education** s**tatus**	Illiterate	18	7.2
	Primary school	74	29.5
	High school	49	19.5
	Associate degree	11	4.4
	University	99	39.4
**Monthly** i**ncome** s**tatus of** y**our** f**amily**	Poor	22	8.8
	Medium	184	73.3
	Good	45	17.9
**Structure of** y**our** f**amily**	Nuclear	193	76.9
	Extended	58	23.1
**Do** y**ou** h**ave** a c**hronic** d**isease?**	Yes	202	80.5
	No	49	19.5
**Are** t**here** a**ny** m**edications** y**ou** u**se** c**onstantly?**	Yes	193	76.9
	No	58	23.1
**n = 251**			

**Table 2:** The goodness of fit values in CFA.

Index values	Normal value	Acceptable value	Model values
x2/sd	<2	<5	44.17/17 = 2.59
GFI	>0.95	>0.90	0.96
AGFI	>0.95	>0.90	0.91
CFI	>0.95	>0.90	0.97
RMSEA	<0.05	<0.08	0.076
RMR	<0.05	<0.08	0.047
NFI	>0.95	>0.90	0.95

**Table 3:** Item correlation analysis of the Engagement in Healthy Ageing Scale.

	Adjusted total question correlation	Cronbach alpha when question was erased	Cronbach alpha
**1. I listen to my body to adapt to daily life**.	0.432	0.791	0.800
**2. I feel happy when I can control my health**.	0.450	0.797	
**3. I have become my doctor over the years**.	0.377	0.796	
**4. I have got plans to make me feel good**.	0.573	0.767	
**5. I do whatever needs to be done for my health**.	0.636	0.758	
**6. My health is under my control**.	0.555	0.770	
**7. I think about things that make me feel good daily**.	0.555	0.770	
**8. I encourage people I care about to live healthy lives**.	0.520	0.776	

**Table 4:** Context validity of the Engagement in Healthy Ageing Scale.

		1	2
1. Engagement in Healthy Ageing Scale			
**2. Health** Seeking Behaviour Scale	r	0.350^**^	
	p	0.000	
**3. Self-**Efficacy Scale	r	0.343^**^	0.034
	p	0.000	0.593

n = 251, p < 0.001^**^.

**Appendix:** Turkish Version of the Engagement in Healthy Ageing Scale [Sağlıklı Yaşlanmaya Katılım Ölçeği Türkçe Versiyonu].

	Sağlıklı yaşlanmaya katılım ölçeği	Kesinlikle katılmıyorum	Katılmıyorum	Kararsızım	Katılıyorum	Kesinlikle Katılıyorum
**1**	Günlük yaşama uyum sağlamak için vücudumu dinlerim.	1	2	3	4	5
**2**	Sağlığımı kontrol edebildiğim zaman mutlu olurum.	1	2	3	4	5
**3**	Yıllar geçtikçe kendi kendimin doktoru oldum.	1	2	3	4	5
**4**	Kendimi iyi hissettirecek yaşam planlarım var.	1	2	3	4	5
**5**	Sağlığım için yapılması gereken neyse onu yaparım.	1	2	3	4	5
**6**	Sağlığım kontrolüm altında.	1	2	3	4	5
**7**	Günlük olarak kendimi iyi hissettirecek şeyler düşünürüm.	1	2	3	4	5
**8**	Önemsediğim insanları sağlıklı yaşam için teşvik ederim.	1	2	3	4	5

## Footnotes

1 World Health Organization (2015). World Report on Ageing and Health [online]. Website https://apps.who.int/iris/bitstream/handle/10665/186463/9789240694811_eng.pdf?sequence=1. [accessed 28.01.2021].
